# Insights into the expression of DNA (de)methylation genes responsive to nitric oxide signaling in potato resistance to late blight disease

**DOI:** 10.3389/fpls.2022.1033699

**Published:** 2022-12-02

**Authors:** Andżelika Drozda, Barbara Kurpisz, Yufeng Guan, Magdalena Arasimowicz-Jelonek, Jarosław Plich, Przemysław Jagodzik, Daniel Kuźnicki, Jolanta Floryszak-Wieczorek

**Affiliations:** ^1^ Department of Plant Physiology, Faculty of Agronomy, Horticulture and Bioengineering, Poznań University of Life Sciences, Poznań, Poland; ^2^ Department of Plant Ecophysiology, Faculty of Biology, Adam Mickiewicz University in Poznań, Poznań, Poland; ^3^ Plant Breeding and Acclimatization Institute - National Research Institute, Młochów, Poland

**Keywords:** nitric oxide, DNA (de)methylation genes, *R3a*, RdDM pathway, *Phytophthora infestans*

## Abstract

Our previous study concerning the pathogen-induced biphasic pattern of nitric oxide (NO) burst revealed that the decline phase and a low level of NO, due to S-nitrosoglutathione reductase (GSNOR) activity, might be decisive in the upregulation of stress-sensitive genes *via* histone H3/H4 methylation in potato leaves inoculated with avr *P*. *infestans*. The present study refers to the NO-related impact on genes regulating DNA (de)methylation, being in dialog with histone methylation. The excessive amounts of NO after the pathogen or GSNO treatment forced the transient upregulation of histone *SUVH4* methylation and DNA hypermethylation. Then the diminished NO bioavailability reduced the SUVH4-mediated suppressive H3K9me2 mark on the *R3a* gene promoter and enhanced its transcription. However, we found that the *R3a* gene is likely to be controlled by the RdDM methylation pathway. The data revealed the time-dependent downregulation of the *DCL3*, *AGO4*, and *miR482e* genes, exerting upregulation of the targeted *R3a* gene correlated with *ROS1* overexpression. Based on these results, we postulate that the biphasic waves of NO burst in response to the pathogen appear crucial in establishing potato resistance to late blight through the RdDM pathway controlling *R* gene expression.

## Introduction

Nitric oxide is an endogenous bioactive signaling molecule associated with various physiological and pathophysiological effects (Del Castello et al., 2019; Kolbert et al., 2019). Nitric oxide is a relatively stable, uncharged molecule and highly diffusible through biological membranes. Although it has a short biological half-life (max. a few seconds) and usually acts locally in the cell compartments, it is also possible to transfer NO bioactivity into the cell nucleus (Wurm and Lindermayr, 2021). NO belongs to the group of redox-signaling molecules. Most of the biological functions of NO are mediated by several mechanisms concerning either its direct or indirect reaction with the iron centers in heme-containing proteins or *via* the formation of protein adducts containing nitrogen oxide through the process of S-nitrosation, transnitrosation, tyrosine nitration or dinitrosyliron complex formation (Gupta et al., 2020; Lindermayr et al., 2020). New findings have recently demonstrated that apart from pleiotropic functions recognized so far; NO reactivity might also be engaged in epigenetic processes involved in histone modifications and DNA methylation in plants (Mengel et al., 2017; Ageeva-Kieferle et al., 2021; Rudolf et al., 2021; Drozda et al., 2022).

Peroxynitrite (ONOO^-^) is a potent oxidant and nitrating species generated by the reaction of NO and superoxide, commonly known as a mediator of cellular injury in many biological systems (Arasimowicz-Jelonek and Floryszak-Wieczorek, 2011; Vandelle and Delledonne, 2011). An increasing number of studies have reported that peroxynitrite may not be considered only as a cytotoxic agent but might also act as a potent modulator of the redox regulation in various cell signal transduction pathways, including pathogen resistance (del Río, 2015; Vandelle et al., 2016; Arasimowicz-Jelonek and Floryszak-Wieczorek, 2019).

A majority of the *R* genes encode the intracellular nucleotide-binding domain and leucine-rich repeat receptors (NB-LRR), which can recognize pathogen effectors and activate rapid and robust effector-triggered immunity (ETI) against the pathogen, involving hypersensitive response (Cui et al., 2015; Jones et al., 2016; Zhou and Zhang, 2020). The expression of the *R* genes is regulated in a precise and multifaceted manner at transcriptional and post-transcriptional levels or by non-coding small RNAs. Small RNAs, including siRNA and miRNA, are involved in several biological processes, including regulating gene expression or silencing transposable elements (Fuso et al., 2020).

In *Solanaceous* species, such as potato and tomato, some miR482, miR5300, miR6019, and miR6027 were identified to target the NB-LRR genes, respectively (Li et al., 2012; Shivaprasad et al., 2012; Cui et al., 2014; Seo et al., 2018). Small RNAs are mobile to exert systemic effects over a long distance within the plant, causing posttranscriptional modifications (PTMs) and other epigenetic changes (Molnar et al., 2010). Generally, the experimental data revealed that miRNAs suppress a wide range of *R* genes that confer resistance to various pathogens; however, the specific mechanism of this regulation seems to vary significantly between species (Shivaprasad et al., 2012; Ouyang et al., 2014; Fei et al., 2016). Precise regulation of *R* genes is pivotal to preventing fitness costs and autoimmune responses in the absence of the pathogen. However, in the presence of an aggressor, early and rapid overexpression of *R* genes is necessary for improved resistance to stress.

In the present paper, special attention is given to the issue of how or whether NO influences the expression of *R* genes implicated in the regulation through the RNA-directed DNA methylation pathway (RdDM). Under changing circumstances, the current state of the 5-mC DNA pattern is often the effect of the cooperation or competition of DNA methyltransferases and the RdDM pathway with DNA demethylation machinery. *De novo* DNA methylation is established by chromomethylase 3 (CMT3), CMT2 for CHG methylation, and domain rearranged methyltransferase 2 (DRM2) for CHH methylation (where H corresponds to A, T, or C). In turn, methyltransferase 1 (MET1) is required for global cytosine methylation maintenance in the CG sequence context. Methyl groups can also be removed from DNA through a DNA base excision repair pathway mediated by 5-methylcytosine DNA glycosylases in *Arabidopsis*, such as repressors of silencing 1 (ROS1), DEMETER (DME), and DEMETER-LIKE 2 (DML2) and DML3. DNA methylation is functionally linked to H3K9me2 through CMT2/CMT3 or DRM2 engaged in the RdDM machinery. Several essential enzymes of the RdDM pathway were detected, including Dicer-like 3 (DCL3), which processes double-stranded RNA to form 24-26-nucleotide siRNAs, or Argonaute 4 (AGO4). AGO4-bound siRNAs interact with Pol V to recruit DRM2 and catalyze *de novo* DNA methylation at CG and non-CG contexts at the homologous genomic sites, affecting Transposon Elements (TEs) and transcriptional gene silencing (Saze et al., 2012; Matzke and Mosher, 2014; Zhang et al., 2018).

Few reports have been published on the potential NO-dependent effect on DNA methylation in plants. It was previously found that seedlings of two *Oryza sativa* L. ssp. Japonica cultivars treated with high doses of sodium nitroprusside (NO donor) showed hypomethylation, mainly in the CHG sequence and transcriptional perturbations of chromatin-remodeling genes (Ou et al., 2015). Recently, it was demonstrated that GSNO reductase-deficient *Arabidopsis* (*gsnor1*-*3*) with enhanced NO levels revealed hypermethylation of TEs and impaired stress-responsive genes (Rudolf et al., 2021). Moreover, the authors proposed that S-nitrosoglutathione reductase (GSNOR) activity is required to control transmethylation cell activity linked with DNA (de)methylation associated with stress-responsive gene regulation.

Methylation is directly linked to S-adenosylmethionine (SAM) acting as a universal methyl (-CH3) donor in a broad spectrum of biological functions, including DNA and histone methylation. Each transfer of the -CH3 group to a methyl acceptor generates S-adenosylhomocysteine (SAH), a competitive inhibitor of methyltransferases that is subsequently cleaved to adenosine and homocysteine using an S-adenosylhomocysteine hydrolase (SAHH). Homocysteine is then converted through methionine to S-adenosylmethionine (SAM), which acts again in this cycle as a methyl donor for methyltransferases in the transmethylation reactions of various acceptors. Growing evidence highlighted the critical role of SAHH in maintaining the methylation potential in this recycling mechanism by regulating the cellular SAM/SAH ratio for DNA and H3K9me2 methylation under physiological or pathological conditions (Palmer and Abeles, 1979; Rahikainen et al., 2018; Saravana Kumar et al., 2020).

In the past, numerous proteome-wide analyses reported that some components involved in the SAM/SAH cycle underwent S-nitrosation (Lindermayr et al., 2005; Abat and Deswal, 2009; Puyaubert et al., 2014; Hu et al., 2015) or tyrosine nitration (Chaki et al., 2009; Lozano-Juste et al., 2011; Begara-Morales et al., 2013; Arasimowicz-Jelonek et al., 2016) modifying the methylation homeostasis in plant cells.

The cultivated potato is the third most important food crop after rice and wheat and is a major horticultural crop (Devaux et al., 2014). Biotic stresses negatively influence plant growth and development, severely reducing crop yield potential and leading to substantial economic losses. The late blight disease of potato and tomato, caused by the oomycete *P*. *infestans*, generates estimated global annual losses of €12 billion (Arora et al., 2014). Plants are constantly exposed to unfavorable biotic and abiotic cues. Research on epigenetic mechanisms in crop response to stress could be necessary to improve crop adaptation to environmental changes and enhance resistance to pathogens in line with the epi-breeding strategy (Springer and Schmitz, 2017; Varotto et al., 2020; Zhi and Chang, 2021).

The presented study aimed to explore the NO-dependent redox targets that can participate in the antagonistic tuning of the *R* gene expression by complementary miRNA during the potato-avr *P*. *infestans* interaction. Our findings revealed that under excessive NO, the central genes of the RdDM pathway were upregulated (*DCL3*, *AGO4*, *DRM2*, and *miR482e*), which suppressed the *R3a* gene expression. Then, diminished NO bioavailability probably resulted in the reduced inhibitory impact of the *miR482e* gene toward the corresponding *R3a* gene, favoring resistance to *Phytophthora infestans*.

## Materials and methods

### Plant material and cultivation

The plant material consisted of two potato genotypes, i.e., ‘Sarpo Mira’ (*Solanum tuberosum* L. cv. Sarpo Mira) and the breeding line TG 97-411 obtained from the Plant Breeding and Acclimatization Institute collection Research Division in Młochów, Poland. Both ‘Sarpo Mira’ and the breeding line TG 97-411 show high effector-triggered immunity (ETI) resistance with the avirulent *Phytophthora infestans* isolate, resulting in a hypersensitive response (HR). The genotype ‘Sarpo Mira’ is characterized by the pyramidization of *R genes* against *P*. *infestans*. Thus this leads to a high degree of resistance to this pathogen, as the genes identified in this R variant are *R3a*, *R3b*, *R4*, *R8*, and *Rpi-Smira1*. The protoplast of the TG 97-411 tetraploid genotype is the interspecific *S*. *phureja* × *S*. *stenotonum* hybrid, from where the *Rpi*-*phu1* gene was introduced into the tetraploid *S*. *tuberosum* (Śliwka et al., 2010). Potato explants were propagated under sterile conditions by the *in vitro* seedling method. They were cultured for 28 days on solidified MS medium (Duchefa Biochemie B.V. Haarlem, the Netherlands) containing 2% (w/v) sucrose and 10% agar (Murashige and Skoog, 1962). Then the plants were transplanted to sterile soil (universal substrate consisting of natural peat, WOKAS SA, Łosice, Poland) and grown to the leaf stage in a phytochamber with 16 h of light (180 μmol m^-2^s^-1^), FLUORA L18W/77, and L58W/77, OSRAM, Germany) at 18 ± 2°C and 60% humidity for 4 weeks.

### Pathogen culture and inoculation

The avr *Phytophthora infestans* (Mont.) de Bary isolate MP946 (A1 mating type, race 1.3.4.7.10.11) and MP324 (A1 mating type, race 1.2.3.4.5.6.7.8.10.11) were kindly supplied by the Plant Breeding and Acclimatization Institute collection Research Division in Młochów, Poland. After three weeks of growth in pea medium, pH=6.1, the pathogen was passaged at least twice through the tubers. The inoculated tuber slices were incubated in airtight plastic boxes for 7-14 days in the dark at 16°C. The sporangia of *P*. *infestans* were obtained by collecting the aerial mycelium, rinsed with cold distilled water, passed through a sterile sieve, and adjusted to a concentration of 2.5 × 10^5^ sporangia per 1 ml using a hemocytometer. Then the sporangia were incubated at 4°C for 1 h to release the zoospores. Potato plants were inoculated by spraying leaves with a zoospore suspension and kept overnight at 18°C and 80-90% humidity on moist blotting paper in a plastic box covered with glass. Inoculated and control leaves were sprayed with distilled water and transferred to a phytochamber. Samples were collected at 1, 3, 6, 24, and 48 h after inoculation (hpi).

### NO donor and scavenger treatment

The third or fourth compound leave from the base of the intact plant was treated by spraying with nitric oxide donor – 250 μM GSNO (S-nitrosoglutathione; Sigma-Aldrich) or a specific scavenger of NO – 200 µM cPTIO (2-(4-Carboxyphenyl)-4,4,5,5-tetramethylimidazoline-1-oxyl-3-oxide; Sigma-Aldrich), which allowed to estimate the effect of eliminating NO from potato leaves or 250 μM GSH (glutathione; Sigma-Aldrich), respectively. GSH is not responsible for NO generation but acts as a reducing compound compared to oxidizing GSNO under physiological conditions. The leaves were sprayed with 5 ml of the mentioned solutions and placed in an airtight, transparent plastic box. Samples were collected at 1, 3, 6, 24, and 48 h after treatment (h).

### Measurement of nitric oxide generation

Nitric oxide production was measured using cell-permeable NO fluorescent probe - CuFL (the copper (II) complex of FL (2-{2-Chloro-6-hydroxy-5-[2-methylquinolin-8-ylamino)methyl]-3-oxo-3H-xanthen-9-yl}benzoic acid. Cu-FL was freshly prepared by adding a 1:1 FL solution (1 mM) to the copper (II) solution (1 mM), as previously specified by Arasimowicz-Jelonek et al. (2016). The fluorescence intensity of the NO-FL complex was determined with the Perkin Elmer LS Fluorescence Spectrometer 50B (UK) using 488 and 516 nm for excitation and emission. Each value was expressed as relative fluorescence intensity (Int × g^−1^ FW).

Nitric oxide emission from potato leaves was also measured using the NO chemiluminescence analyzer (CLD 88, Eco Physics, Switzerland) as described by Planchet and Kaiser, 2006 and Zafari et al. (2022), with some modifications. The leaf segments (2g FW) from 4-week-old potato plants were treated with the pathogen, 250 µM GSNO, 200 µM cPTIO, or H_2_O, and immediately placed into a measuring glass chamber. NO-free helium gas with a constant flow of 400 mL min^-1^ was first passed through the measuring chamber with potato leaves and subsequently through the NO chemiluminescence analyzer that is sensitive in a range of 0-4000ppb of NO. Microsoft Excel visualized the NO measurement recorded every second for approx. 7 hours. Each value was expressed as NO emission (ppb×g^-1^FW×h^-1^).

### Gene expression analysis

Potato leaf fragments collected at the appropriate time points were frozen in liquid nitrogen and stored at -80°C until use. RNA was then isolated from 100 mg of frozen tissue using TriReagent (Sigma-Aldrich) according to the manufacturer’s protocol. The obtained RNA was then purified using a special Deoxyribonuclease kit (Sigma-Aldrich). Reverse transcription of 1 µg of RNA for each experimental variant was performed using a reverse transcription kit (Thermo Fisher Scientific, USA). RT-qPCR analyses were performed on a PikoReal 96 Thermocycler (Thermo Fisher Scientific, USA) under the following conditions: 10 min at 95°C, followed by 45 cycles of 12 s at 95°C, 30 s at the annealing temperature for each specific primer (**Table S1**) and 30 s at 72°C. The reaction mixture contained 0.1 μM of each primer, 1 μl of 5 × diluted cDNA, 10 μl of the Power SYBR^®^ Green PCR Master Mix (Applied Biosystems, United States), and DEPC treated water to a total volume of 20 μl. Primers for the studied genes were designed using the Primer-blast program by the available NCBI (National Center of Biotechnology Information) and PGSC (Potato Genome Sequencing Consortium) databases. The primers designed and used in this study are listed in **Table S1**. The obtained data were normalized to the elongation factor *ef1α* (AB061263) and *18S rRNA* (X67238). The Ct values were determined using the Real-time PCR Miner (Zhao and Fernald, 2005), and relative gene expression was calculated using efficiency-corrected computational models proposed by Pfaffl (2001) and Tichopad et al. (2004).

### Analysis of miRNA expression – quantitative steam-loop PCR method

The amount of the mature miRNA transcript was determined according to Varkonyi-Gasic and Hellens (2011) and Varkonyi-Gasic (2017). The primers used were designed according to the modified method of Chen et al. (2005) and are given in **Table S2**. Total RNA, also used in gene expression analyses, was reverse transcribed. The 1 μM loop primer solution was denatured at 65°C for 5 minutes and then kept on ice until used. The samples contained 1 μl of total RNA, 4 μl of 5 × concentrated reaction buffer, 0.1 μl of RNase inhibitor (Ribolock RNase inhibitor, 40 U*μl^-1^), 0.5 μl 10 mM dNTP mixture, 0.25 μl of reverse transcriptase (200 U*μl^-1^), 1 μl of denatured loop primers, 1 μl of oligo-dT oligonucleotides and 12.15 μl of RNAse-free water. The samples were lightly centrifuged and placed in a PikoReal 96 thermal cycler (Thermo Fisher Scientific) with the following reaction conditions: 30 minutes at 16°C, followed by 60 cycles of 30°C for 30 s, 42°C for 30 s, and 50°C for 1 s. After this, the reverse transcription process was stopped by heating at 85°C for 5 minutes; samples were cooled at 4°C and diluted 5 times by adding 80 μl of DEPC water.

The level of miRNA transcript was also determined using the PikoReal 96 RT-qPCR instrument (Thermo Fisher Scientific). The volume of the reaction mixture was 20 µl consisting of 12 µl of DEPC water, 2 µl of 100 mM selective primer mix, 2 µl of reverse transcription assay, and 4 µl of polymerase solution (Power SYBR^®^ Green PCR Master Mix, Qiagen). Negative samples had a similar composition, but instead of 2 µl of the test sample, 2 µl of DEPC water was added as a volume equivalent. The thermal profile of the reaction was as follows: initial denaturation at 95°C for 5 minutes, 35 cycles of denaturation at 95°C for 5 s, primer annealing at a specific temperature for 10 s, and an extension step at 72°C for 15 s. The melting curve was obtained by heating the samples from 65°C to 95°C at 0.1°C*s^-1^. The specificity of the amplification was assessed mainly by the analysis of the melting curves of the products. In addition, in the case of the preparation of new primers, electrophoretic analysis of the product length was performed. In the case of the appearance of a non-specific product, the result was not taken into account in further calculations. The results were analyzed using the PCR Miner algorithm provided by Zhao and Fernald (2005). The relative amount of the test miRNA transcript was calculated in relation to the control and the *ef1α* (AB061263) and *18S rRNA* (X67238) reference genes (Nicot et al., 2005), using the Pfaffl method (2001).

### Chromatin immunoprecipitation assay

The chromatin immunoprecipitation assay (ChIP) was carried out as described by Haring et al. (2007) and Komar et al. (2016). The sample of 2 g potato leaves was cross-linked by vacuum infiltration in a crosslinking buffer with 1% formaldehyde and frozen at -80°C. The next step was chromatin isolation, performed according to Jarillo et al.’s protocol (2018) with some modifications. Samples were ground in liquid nitrogen, resuspended, and incubated in Nuclei Isolation Buffer, and after centrifugation, resuspended in Nuclei Lysis Buffer. Subsequently, the samples were sonicated on ice for 30 × 30 s at 30% power to obtain DNA fragments of 250-750 nt in length. An input sample (50-100 μl) was collected from the solution to check the quality of the model on an agarose gel. The remaining solution was separated into the test sample (to which the antibody of interest was added: H3K9me2 (EMD Millipore; cat.-no. 07-411) and the control sample (to which IgG was added). The next day 30 μl of Magnetic Beads (PureProteome Protein A/G Mix, Millipore) were added, and the samples were incubated for at least 2 hours. Afterward, the samples were washed and decrosslinked overnight with 300 mM NaCl and 1% SDS at 65°C with shaking. The next step involved incubating probes with proteinase K (20 mg/ml) to digest proteins. Then the samples were subjected to DNA isolation with phenol: chloroform: isoamyl alcohol mixture (25:24:1). The last step was to check the number of binding sites in the immunoprecipitated DNA using the RT-qPCR method. The reaction mixture contained 0.1 μM of each primer, 2-5 μl of purified DNA, 10 μl of Power SYBR^®^ Green PCR Master Mix (Applied Biosystems), and DEPC treated water to a total volume of 20 μl. The specificity of the reaction was confirmed by the presence of one peak in the melting curve analysis. Primers for the gene of interest (*R3a*) were designed by Primer3 Output Software (**Table S3**). Data were analyzed by the Fold Enrichment Method (Jarillo et al., 2018). The raw Ct value of each sample was subtracted from the raw Ct value of the control (IgG) corresponding to that sample (ΔCt=Ct_(sample)_-Ct_(control, IgG)_ ). The enrichments were calculated using the following formula:


$${Fold enrichment=2}^{-\Delta \text{Ct}}$$

After treatment with GSNO (250 μM), cPTIO (200 μM), and GSH (250 μM), and after avr *P*. *infestans* inoculation, samples were taken at 3, 6, and 24 h. The relative amount of immunoprecipitated chromatin fragments (as determined by real-time PCR) from the above treatment variants was compared with the reference (arbitrarily set to 1). The reference (leaves sprayed with water) was taken at each time point.

Each experiment included at least three independent measurements per sample. The P values for each sample combination were calculated using ANOVA. The Tukey-Kramer test compared the mean values (α=0.05 and α=0.01).

### ELISA test for global 5-mC DNA

DNA isolation was performed using the phenol-chloroform method, with 150 mg of an aliquot of leaves frozen in liquid nitrogen (Sambrook and Russell, 2006). 2 μl of proteinase K and 1 μl of RNase (Sigma-Aldrich) were added to the plant material homogenized in 1 ml of TE buffer (10 mM Tris-HCl, 10 mM EDTA, 100 mM NaCl, 2% SDS), the whole was incubated at 37°C for 30 minutes and then at 95°C for 5 minutes to stop the reaction. After adding a 1:1 v/v mixture of phenol and chloroform, the samples were vigorously shaken and then centrifuged for 12 minutes at 4°C (10000 × g). The upper phase was collected in a new tube, and 400 µl of a mixture of chloroform and isoamyl alcohol (2:1 v/v) was added, followed by repeated shaking and centrifugation for 12 minutes at 4°C (10000 × g). The upper phase was harvested again, and 180 µl of isopropanol was added, with the samples incubated for 10 minutes at room temperature and centrifuged for 12 minutes at 4°C (10000 × g). The supernatant was discarded, and the pellet was left for 10 minutes in open tubes at 25°C to dry. After this time, 30 μl of DEPC water (Bioshop) was added to the tests. The quality control of the obtained DNA was performed both by the electrophoretic method and quantitative and qualitative measurements on the NanoDrop 2000 device (Thermo Fisher Scientific). The samples were diluted with DEPC water to a concentration of 0.1 µg DNA*µl^-1^ of the solution and then frozen at -20°C, thus storing them for further analysis.

According to the manufacturer’s protocol, global 5-mC detection was performed with the 5-mC ELISA kit (Zymo Research, United States). 100 ng of each probe was added to a PCR tube and brought to 100 μl with 5-mC Coating Buffer. Next, all DNA samples were denatured for 5 minutes (98°C) and immediately transferred to ice. After cooling, samples were added to the wells on the plate, covered with foil, and incubated at 37°C for 1 hour. After incubation, the probes were washed and blocked in 5-mC ELISA Buffer. The next step was the addition of the antibody. 100 μl of the antibody mix containing Anti-5-Methylocytosine and Secondary Antibody were added. Plates were incubated at 37°C for 1 hour. After that time, each well was washed 3 times with 5-mC ELISA Buffer. In the last step, 100 μl of HRP Developer was added to each well and allowed to develop color for 10-60 minutes in RT. Absorbance was read at 450 nm using an ELISA plate reader (Tecan, Infinite F50 Plus). The percentage of 5-mC was calculated using the following formula:


$${\% 5m-C=e}^{\left\{\frac{\text{Absorbance-y-intercept}}{\text{Slope}}\right\}}$$

### Protein immunoprecipitation

Protein immunoprecipitation was performed according to the protocol described by Zhao et al. (2017). Potato leaves (0.75 g) were ground to a fine powder in liquid nitrogen. The powder was suspended in 375 µl of binding buffer (50 mM Tris-HCl [pH 8.0], 100 mM NaCl, 1 mM EDTA, 0.5% NP-40, 1% protease inhibitor cocktail (Merck Group, Darmstadt, Germany). Homogenates were centrifuged at 16000 × g for 10 min, and the supernatants were transferred to the fresh tube. The protein concentration was measured by the Bradford assay with bovine serum albumin (BSA) as the standard protein (Bradford, 1976). Protein samples (2.5 mg) were incubated overnight with an anti-nitrotyrosine polyclonal antibody (Thermo Fisher Scientific, Waltham, Massachusetts, United States) at 8 µg/1 mg of protein at 4°C with gentle rotation in a total volume of 450 µl. Simultaneously, protein samples used as the negative control were incubated without antibodies. After incubation, 400 µl of 50% protein G beads (Merck Group, Darmstadt, Germany) (in PBS buffer) were added to the solution for overnight incubation at 4°C with gentle rotation. The supernatants (unbound proteins) were removed by carefully washing the beads once with 2.5 ml of binding buffer, twice with 2.5 ml of washing buffer 1 (50 mM Tris HCl [pH 8.0], 100 mM NaCl, 1 mM EDTA), once with 2.5 ml of washing buffer 2 (50 mM Tris [pH 8.0], 100 mM NaCl, 1 mM EDTA, 10% acetonitrile [ACN]) and once with 2.5 ml of water. Nitrotyrosine-containing proteins were eluted from the beads with 1 ml of low-pH acetonitrile solution (0.5% TFA, 25% ACN) and collected in 10 fractions. The most protein-abundant fractions were selected based on SDS-PAGE analysis and combined. The protein concentration was calculated based on SDS-PAGE analysis by summing the intensity of the pixels within each protein band image with BSA as the standard protein.

### Western blot

Equal amounts (0.2 µg) of nitrotyrosine-containing proteins were incubated with sample buffer (62.5 mM Tris–HCl, pH 8.5, 10% sucrose, 2% SDS, 0.025% bromophenol blue, 0.1 M dithioerythritol) at 95°C for 3 min and separated by standard SDS-PAGE in 12% polyacrylamide gels, electrotransferred on to a PVDF membrane and immunostained with antibodies against the anti-S-adenosylhomocysteine hydrolase (n-terminal) antibody (Merck Group, Darmstadt, Germany) (1/2000, v/v) in 1% BSA/TBS-T at 4°C overnight. After washing the membrane, it was treated with horseradish peroxidase-conjugated goat anti-rabbit IgG antibody (1/25000, v/v) in TBS-T for 1 h. According to standard procedures, the signals were visualized using the chemiluminescence method and quantified using the Image Lab™ software (Bio-Rad; Hercules, CA, USA). The statistical significance of the differences in signal intensity was analyzed using Student’s *t*-test at *P*<0.05.

### Statistical analysis

All the experiments included three independent experiments in at least three replications. For each experiment, the means of the obtained values were calculated along with standard deviations. The analysis of variance was conducted, and the least significant differences (LSDs) between means were determined using Tukey’s test at the levels of significance α=0.05 (^∗^) and α=0.01 (^∗∗^). Statistical analyses were performed using Microsoft Excel 2016 and R statistical software (version 4.1.2).

## Results

### Time-dependent NO overproduction after pathogen or GSNO treatment

**Figure 1:**
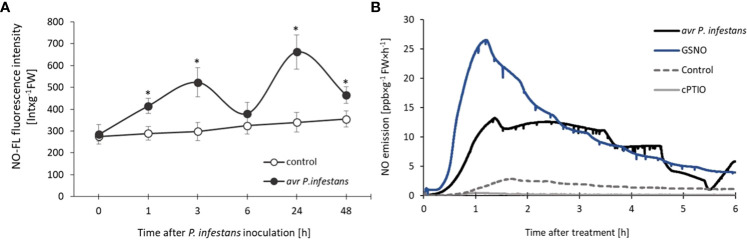
Nitric oxide generation in potato leaves of resistant ‘Sarpo Mira’ challenge inoculated with avr *P*. *infestans* measured by fluorescence method **(A)**, NO emission from leaves treated with the pathogen, 250 µM GSNO, 200 µM cPTIO, or H_2_O, measured by chemiluminescence using an ultra-high selective NO analyzer **(B)**. In **(A)** NO-FL fluorescence from extracts of control and inoculated potato leaves was determined as described in the Materials and methods. Each value was expressed as NO-FL fluorescence intensity in Int × g^-1^ FW. Values represent the means of data ± SD of three independent experiments. Asterisks indicate values that differ significantly from those for control leaves at α<0.05 (^∗^). In **(B)** chemiluminescence rate of NO emission was expressed in ppb × g^-1^ FW × h^-1^.

The experiments were carried out on the potato leaves of two resistant cultivars giving an HR-type response with a corresponding avr *Phytophthora infestans* isolate and analyzed NO-mediated changes in the early hours after inoculation, decisive for plant immunity. In agreement with our previous findings (Drozda et al., 2022), a pathogen-induced biphasic NO burst was shown. We identified two waves of NO overproduction consisting of an initial sharp increase (at 3 hpi), subsequent decline (at 6 hpi), and a second (at 24 hpi) pronounced phase of NO generation (**Figure 1A**).

Nitric oxide emitted from potato leaves treated with 250 µM GSNO was even twice higher than from avr *P*. *infestans* inoculated leaves during the first two hours after the treatment, measured by the chemiluminescence method (**Figure 1B**). However, the initially high NO level from GSNO diminished temporarily, in contrast to the second re-increase (at 6 hpi) after the pathogen challenge, which is also observed using the cell-permeable NO fluorescent probe – CuFL (**Figure 1A**). Control or H_2_O-treated leaves showed low basal NO production, and 200 µM cPTIO scavenged endogenous NO in potato leaves (**Figure 1B**).

### Enhanced NO level promotes transient upregulation of histone methyltransferase SUVH4-mediated H3K9me2 and DNA hypermethylation

**Figure 2:**
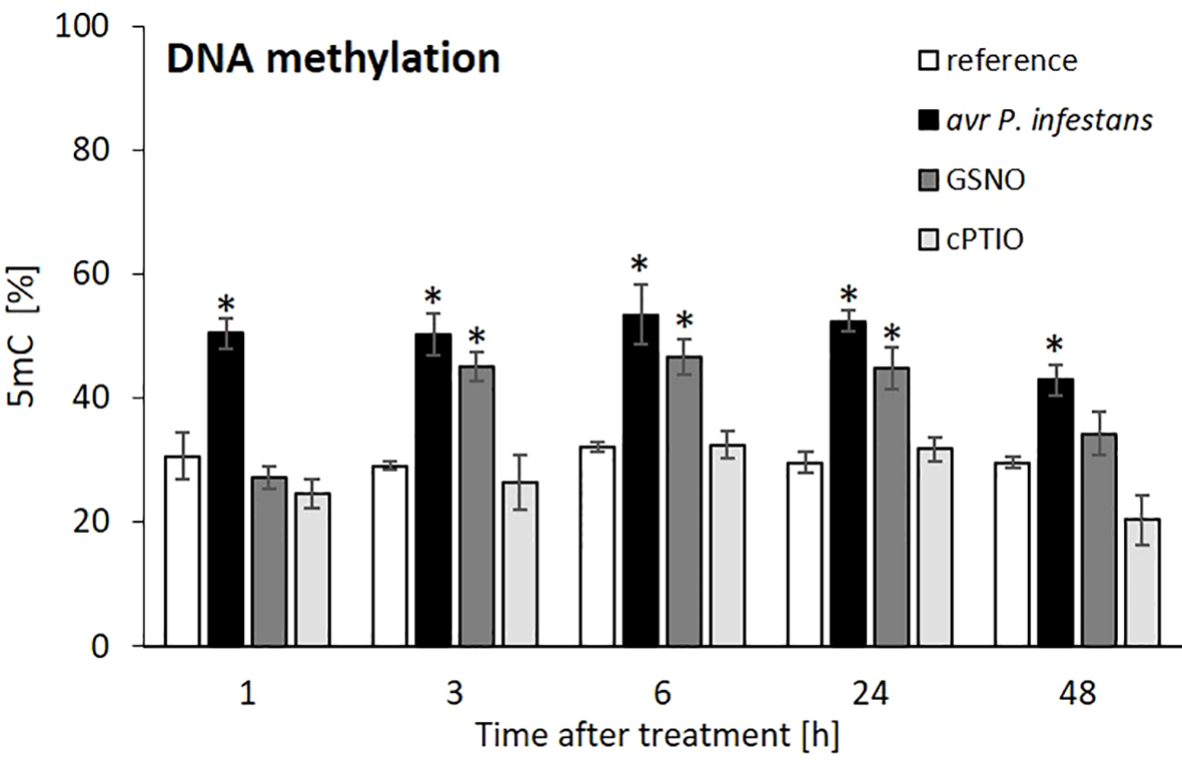
Effect of avr *P. infestans* or GSNO on global DNA methylation in potato leaves of cv. Sarpo Mira. The ELISA test of 5-mC DNA level was performed at selected time points at 1-48 h after GSNO, cPTIO treatment, or challenge inoculation. There were no significant changes in the absolute values of the analyzed 5-mC levels after the leaves spraying with water.Values represent the means of data ± SD of at least three independent experiments. Asterisks indicate values that differ significantly from water-treated (reference) potato leaves at α<0.05 (^∗^).

To investigate the global DNA methylation status, we performed an ELISA assay and found that the level of 5-mC DNA significantly increased after inoculation. The pathogen triggered an almost 2-fold enrichment of 5-mC levels from 1 to 48 hpi (**Figure 2**). Similarly, GSNO treatment heightened DNA methylation from 3 to 24 h compared to cPTIO, respectively (**Figure 2**). These data indicate a rise in NO-mediated DNA global hypermethylation (growth to 50-55%).

Also, TG-line showed a significant increase in 5-mC level after avr *P*. *infestans* or GSNO treatment, similarly to the cv. Sarpo Mira (Supplementary Figure S1). Our results showed that elevated NO production (from endogenous or exogenous sources) resulted in DNA hypermethylation in two resistant potato cultivars in response to avr *P*. *infestans* or GSNO.

The CMT3 chromomethylase is required to maintain DNA methylation preferentially in the CHG context. The CMT3 activity depends on the H3K9me2 mark driven by SUVH4/KYP methyltransferase to guide DNA methylation (Pikaard and Scheid, 2014).

**Figure 3:**
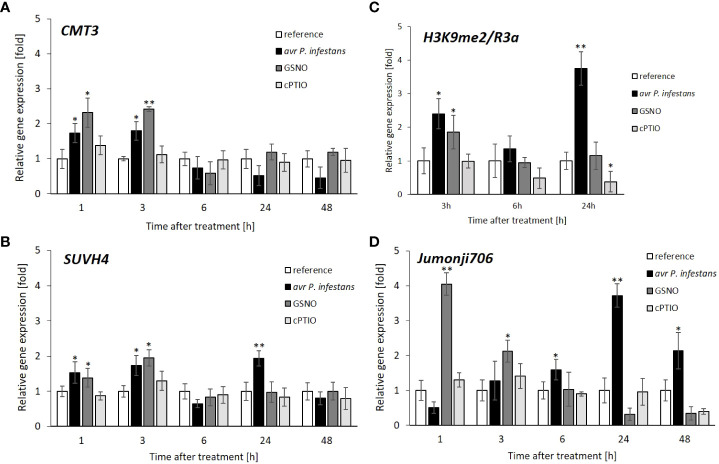
Functional link between histone and DNA methylation due to potato leaves treatment with pathogen or GSNO. *CMT3* DNA methyltransferase expression profile **(A)**, *SUVH4* histone methyltransferase expression profile **(B)**, distribution levels of SUVH4/mediated H3K9me2 on the promoter sequence of *R3a* **(C)**, *JMJ706* histone demethylase expression profile **(D)**. RT-qPCR gene expression of *CMT3*, *SUVH4*, and *JMJ706* was analyzed in potato leaves (at 1-48 h) after treatment with GSNO, cPTIO, water, or avr *P*. *infestans* inoculation, respectively. ChIP-qPCR analyses were performed in potato leaves at selected time points (3-24 h) after treatment with GSNO, cPTIO, water, or avr *P*. *infestans* inoculation. Data are presented as X-fold enrichment (Komar et al., 2016). The relative amount of immunoprecipitated chromatin fragments (as determined by real-time PCR) from the above treatment variants were compared with the reference (arbitrarily set to 1). There were no significant changes in the absolute values of the analyzed transcript levels after the leaves spraying with water. Each experiment included at least three independent measurements per sample. *P* values for each sample combination were calculated using ANOVA, and mean values were compared using the Tukey-Kramer test (α=0.05 (^∗^) and α=0.01 (^∗∗^)).

The obtained data concerning *CMT3* gene expression revealed approx. a 2-fold increase (at 1-3 h) correlated in time with the first peak of biphasic NO overproduction after the pathogen challenge (**Figure 1** and **Figure 3A**). Interestingly, the *SUVH4* expression displayed a similar transcriptional profile as *CMT3*, manifested in a transient increase of gene expression at 1-3 h in response to pathogen inoculation or GSNO exposure (**Figure 3B**). We also found enrichment of *SUVH4*-mediated H3K9me2 mark at 3 h on the promoter region of the *R3a* gene after the avr *P*. *infestans* challenge or GSNO (**Figure 3C**). Next, the same region of the promoter was analyzed at 6 h. The results showed that a decrease in H3K9me2 mark deposition positively correlated with the time-dependent downregulation of *SUVH4* during the same period.

The JMJ706 has been documented as a histone demethylase, precisely removing H3K9me2, thus disassembling heterochromatin from the repressive state (Sun and Zhou, 2008; Qian et al., 2015). The results exhibited different trends in the JMJ706 transcript profile for GSNO and the pathogen. The data showed that JMJ706 transcription first drastically increased (a 4-fold growth) and then gradually diminished in the following hours after GSNO treatment (**Figure 3D**). In contrast, *JMJ706* gene expression gradually increased up to 24 hpi and then decreased slightly in potato leaves challenged with avr *P*. *infestans* (**Figure 3D**). Thus, these results indicate that the transcriptional changes of JMJ706 histone demethylase occur largely independently in response to GSNO or the pathogen.

### Pathogen downregulates *SAHH* gene expression and SAHH protein undergoes Tyr-nitration

Given the role of SAHH in maintaining methylation homeostasis and that the CMT3 pathway is uniquely sensitive to SAHH impairment (Mull et al., 2006), we further focused on SAHH transcriptomic and post-transcriptomic changes after the pathogen inoculation or GSNO exposure.

**Figure 4:**
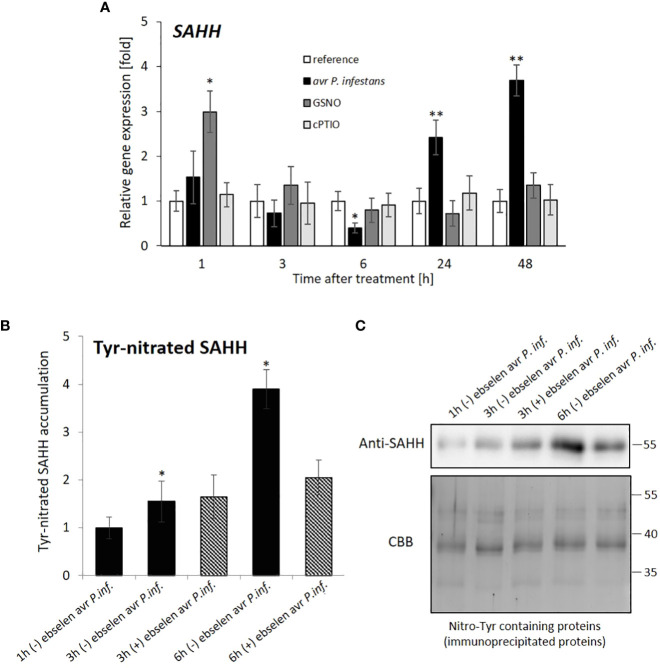
*SAHH* hydrolase expression profile **(A)** and Tyr-nitration of SAHH in potato leaves **(B, C)**. RT-qPCR gene expression of *SAHH* was analyzed in potato leaves (at 1-48 h) after treatment with GSNO, cPTIO, water, or avr *P*. *infestans* inoculation, respectively. There were no significant changes in the absolute values of the analyzed SAHH transcript levels after the leaves spraying with water. For SAHH tyrosine nitration, potato leaves were inoculated with avr *P*. *infestans* in the presence (+) or absence (-) of ebselen (nitrating agent scavenger). A representative SDS-PAGE and Western blot of immunoprecipitated nitroTyr-containing proteins probed with a polyclonal antibody against S-adenosylhomocysteine hydrolase (SAHH) diluted at 1:2000. Protein extracts of *S. tuberosum* leaves were immunoprecipitated with an antibody against nitroTyr. The resulting immunoprecipitated proteins (0.2 µg per lane) were separated by one-dimensional SDS-PAGE in duplicate, and either Coomassie Brilliant Blue (CBB) stained or electrotransferred onto a PVDF membrane and probed with antibodies against SAHH. Asterisks indicate values that differ significantly from water-treated (reference) potato leaves at α<0.05 (^∗^) and α<0.01 (^∗∗^).

The *SAHH* gene expression was transiently downregulated markedly at 6 hpi, and then increased after the pathogen challenge (**Figure 4A**). Western analyses confirmed that avr *P*. *infestans* induced SAHH tyrosine nitration, mainly at 6 hpi, which correlated with the time of SAHH transcript inhibition (**Figures 4A-C**). In turn, GSNO application caused an early increase, followed by the *SAHH* expression return to the baseline level (**Figure 4A**).

Data indicate that a drastic *SAHH* inhibition at 6 h after GSNO or pathogen treatment correlated with transcriptional inhibition of CMT3 functionally linked to SUVH4 mediated by H3K9me2 mark deposition. At a later stage (at 24-48 h), only the pathogen provoked *SAHH* and *JMJ706* gene expression to rise again.

### Potato *R3a* gene is likely to be controlled by the RdDM pathway

**Figure 5:**
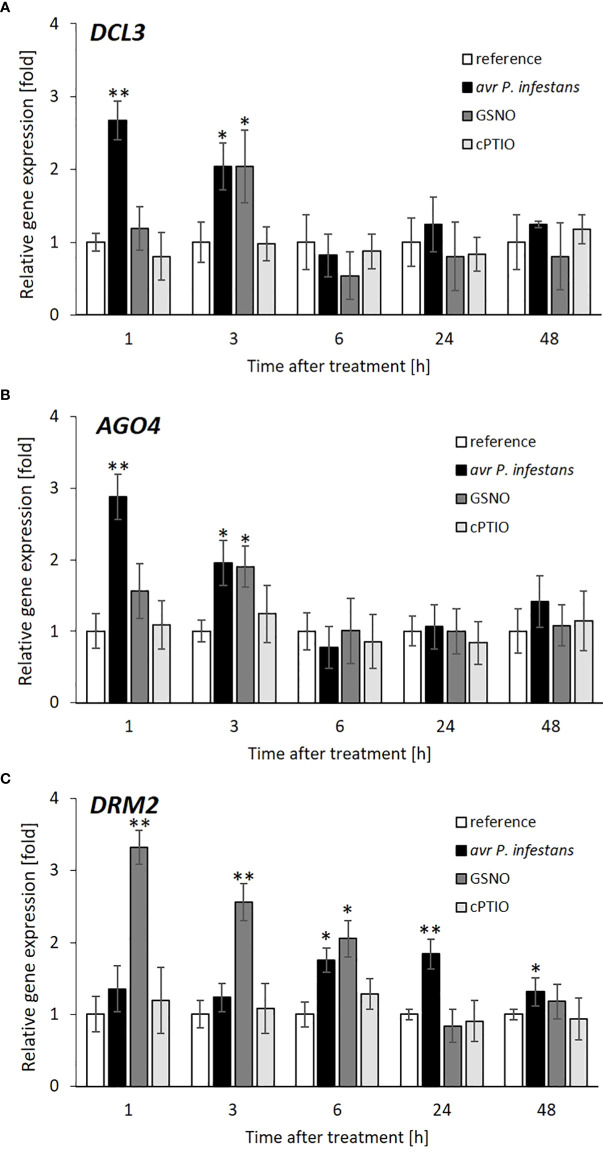
Effect of avr *P*. *infestans* or GSNO on RdDM pathway. RT-qPCR analysis of the *DICER* (*DCL3*) **(A)**, *ARGONAUTE* (*AGO4*) **(B)** domains rearranged methyltransferase 2 (*DRM2*) **(C)**, respectively, were performed at selected time points at 1-48 h after GSNO, cPTIO treatment or challenge inoculated. There were no significant changes in the absolute values of the analyzed transcript levels after the leaves spraying with water. Values represent the means of data ± SD of at least three independent experiments. Asterisks indicate values that differ significantly from water-treated (reference) potato leaves at α<0.05 (^∗^) and α<0.01 (^∗∗^).

To determine how NO-mediated signaling or transnitrosation processes affect the RdDM pathway, the expression of two essential genes of this pathway, *DICER* (*DCL3*) and *ARGONAUTE* (*AGO4*), was examined. The time-dependent analysis of *DCL3* and *AGO4* expression revealed a drastic increase (approx. 3-fold) at 1 hpi, followed by a gradual decrease until 6 hpi in response to the pathogen challenge (**Figures 5A, B**). Interestingly, *DCL3* and *AGO4* transcription tended to rise later (at 24-48 hpi) but only after inoculation. Similar growth (a 2-fold increase) of transcriptional levels for *DCL3* and *AGO4*, mainly at 3 h, was found after GSNO treatment (**Figures 5A, B**).

The domains rearranged methyltransferase 2 (DRM2) responsible for *de novo* DNA methylation in tandem with RdDM components was differentially expressed in the case of both treatments. The pathogen weakly affected *DRM2* gene expression, except for a transient upregulation (up to a 2-fold increase), mainly at 6 and 24 hpi (**Figure 5C**). In turn, GSNO initially (at 1 h) induced a drastic increase (more than 3-fold) of the mRNA transcript level for *DRM2*, which gradually decreased in the following time points after the treatment.

It was well documented that the miR482 family regulates gene expression of target mRNA associated with silencing the *R3a* gene (Li et al., 2012; Shivaprasad et al., 2012). The potato *R3a* gene encodes key immune leucine-rich repeat receptors, which overexpression triggered HR-type immunity to late blight (Kuźnicki et al., 2019). Therefore the challenge was understanding how NO-enhancing potato resistance might affect miRNA and *R* gene interaction.

**Figure 6:**
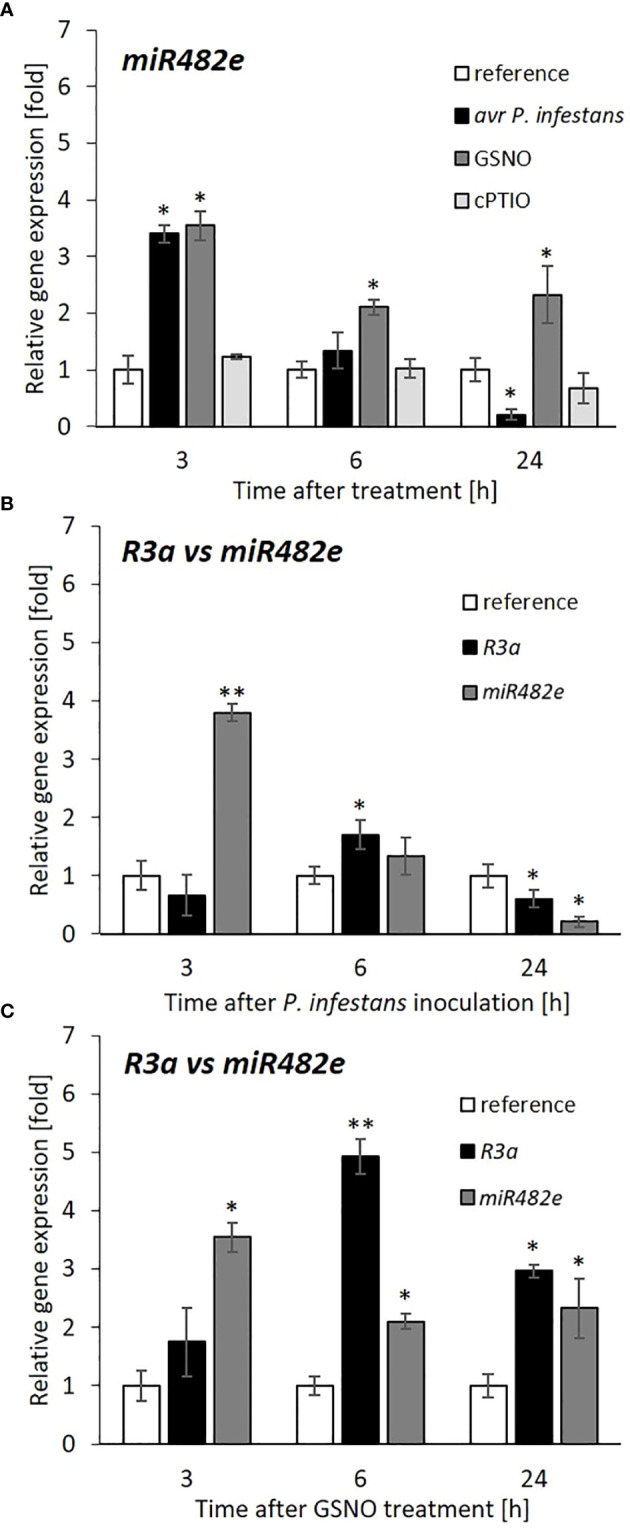
Expression profile of *miR482e* and its target, *R3a* gene after avr *P*. *infestans* or GSNO treatment. RT-qPCR analysis of the *miR482e* **(A)**, relative *miR482e* versus *R3a* genes expression after pathogen **(B)**, or GSNO **(C)** treatment, respectively. All data in **(B)** and **(C)** regarding *R3a* and *miR482e* transcript levels referred to the separate reference presented as 1 for *R3a* and *miR482e*, respectively. Values represent the means of data ± SD of at least three independent experiments. Asterisks indicate values that differ significantly from water-treated (reference) potato leaves at α<0.05 (^∗^) and α<0.01 (^∗∗^).

Transcriptional profiling of *miR482e* revealed a time-dependent upregulated transcript level (a 3.5-fold increase) at 3 hpi, which markedly decreased in the following 6-24 h after the pathogen challenge or GSNO treatment when compared to cPTIO (**Figure 6A**).

Interestingly, a study on target *R* gene expression showed enhanced mRNA transcript accumulation for the *R3a* gene at 6 hpi after inoculation (**Figure 6B**). Also, upon GSNO application, the *R3a* gene expression displayed a similar trend of transcriptional activity peaking at 6 h, which was even more pronounced in response to GSNO than the pathogen (**Figure 6C**). Generally, our data provided evidence that a transient decrease in *miR482e* expression resulted in an increase in *R3a* gene expression at the same time point (6 h) after pathogen or GSNO treatment.

Notably, a similarly time-dependent negative correlation was found between *miR6026* and *Rpi*-*phu1* transcript accumulation in the TG line of potato leaves at 6 h, after GSNO or pathogen challenge (Supplementary Figure S2).

Our study revealed a relationship between elevated NO levels (at 3 h) and upregulated miRNA, suppressing the *R* gene expression. Next, the transient decline of NO generation (at 6 h) simultaneously with downregulated miRNA potentiated the *R* gene transcription. Based on these results, we suggest that the biphasic waves of NO burst in NO-mediated miRNA regulation appear crucial in establishing the late blight resistance to avr *P*. *infestans* by controlling *R* gene expression.

### *ROS1* demethylase contributes to *R3a* upregulation

**Figure 7:**
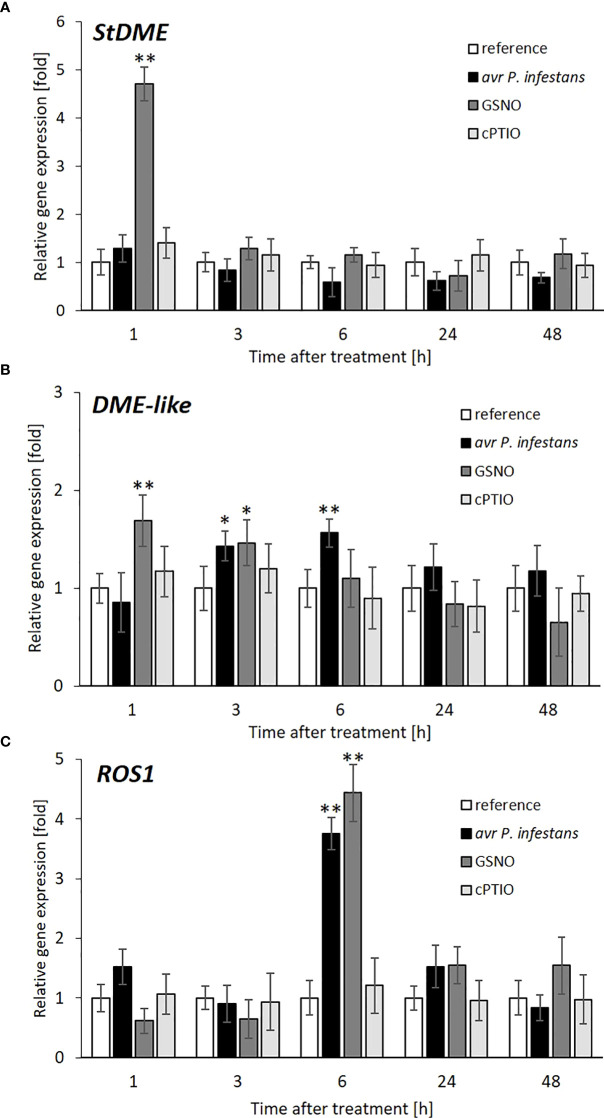
DNA demethylase gene expression after pathogen or GSNO treatment. RT-qPCR analyses of the *StDME* **(A)**, *DME*-*like* **(B)**, and *ROS1* **(C)**, respectively, were performed at selected time points at 1-48 h after GSNO, cPTIO treatment, or challenge inoculation. There were no significant changes in the absolute values of the analyzed transcript levels after the leaves spraying with water. Values represent the means of data ± SD of at least three independent experiments. Asterisks indicate values that differ significantly from water-treated (reference) potato leaves at α<0.05 (^∗^) and α<0.01 (^∗∗^).

Next, we tried to explain whether the previously observed NO-mediated increase of the global 5-mC DNA level was due to the inhibition of demethylation processes; thus, the corresponding mRNA transcript levels for DEMETER (DME) and DEMETER-LIKE (DML) were quantified. The pathogen did not influence *StDME*, while the *DML-like glycosylase* transcript was slightly upregulated in the following time points after inoculation (**Figures 7A, B**). Both *StDME* and *DML-like* genes, except for early upregulation, were also not responsive to the GSNO treatment (**Figures 7A, B**). It is not surprising, given that NO bioactivity might (if any) affect DNA demethylases mainly at the post-translational level.

The *REPRESSOR OF SILENCING 1* (*ROS1*) is activated in vegetative tissue and contributes to stress responses. The expression of *ROS1* is regulated transcriptionally by a complex balance between DNA methylation and demethylation status. Our result indicated a strong association between both treatments regarding *ROS1* transcriptional activity (**Figure 7C**). A significant increase (more than 4-fold) in *ROS1* transcript accumulation was found mainly at 6 h in response to avr *P*. *infestans* or GSNO. Our data suggest that elevated expression of *ROS1* could be a consequence of the enhanced activity of *de novo* methylation of the RdDM pathway found earlier.

### GSH otherwise affects DNA (de)methylation than pathogen or GSNO

To verify NO’s contribution to analyzed processes, leaves were treated independently with reduced glutathione (GSH) as an additional control. Since the reductive decomposition of GSNO might influence GSH formation, and GSH is involved in controlling epigenetic regulation at different levels (Saravana Kumar et al., 2020), we examined how GSH might affect the genes involved in the transcriptional network of DNA (de) methylation. The obtained results are presented in **Supplementary Figures S3A–K**. GSH treatment resulted in a progressive and significant increase in *DRM2 e*xpression up to 24 h and insignificant changes in SAHH and CMT3 transcripts accumulation, related to the reference (**Supplementary Figures S3A–C**). Moreover, GSH did not change *DCL3* and *AGO4* gene expression levels (**Supplementary Figures S3D, E**) and insignificantly altered *StDME* and *DML*-*like* genes transcription compared to controls (**Supplementary Figures S3F, G**). Unexpectedly, GSH treatment induced a progressive increase (up to 3.5-fold) in *ROS1* (**Supplementary Figure S3H**), correlated in time with enhanced *DRM2* gene expression. Histone *SUVH4/KYP* methyltransferase gene expression was significantly upregulated, but the *JMJ706* gene was not responsive to GSH treatment (**Supplementary Figures S3I, J**). Together, these data suggest that GSH treatment could also change DNA (de)methylation homeostasis manifested mainly by increasing *DRM2* methyltransferase and *ROS1* demethylase gene expression. Its effect on the other investigated genes was weaker and different from GSNO. However, the analysis confirmed that GSH did not affect global 5-mC DNA levels in the following hours after the treatment (**Supplementary Figure S3K**).

## Discussion

### Pathogen similarly to GSNO induces potato DNA hypermethylation

Nitric oxide is a multifaceted mediator of plant immunity, exerting numerous effects depending on the kinetics of NO generation, cell localization, local concentration, distribution, and metabolic consumption. In addition, the impact of NO bioactivity on plant pathophysiology is affected by the presence of additional free radicals, their scavengers, and the genetic background of the host or pathogen. Therefore, the NO concentration and temporal-dependent effects determine interactions with different cell targets leading to negative or positive disease outcomes (Thomas, 2015; Sánchez-Vicente et al., 2019).

Our recent findings have provided insights into NO-associated potato immunity to avr *P*. *infestans*, including redox- and time-dependent crosstalk between histone lysine and arginine methylation, which contributes to reprogramming defense genes (Drozda et al., 2022). These data concerning the pathogen-induced biphasic pattern of NO burst revealed that rather the decline phase and a low level of NO due to GSNOR activity might be decisive in facilitating the upregulation of stress-sensitive genes. The present research extends our previous study by examining the NO role in regulating DNA methylation, remaining in dialog with histone methylation in potato immunity to late blight.

Data presented here show that the pathogen rapidly elicited 5-mC DNA hypermethylation in two potato genotypes. A significant increase in the global methylation level was similarly observed for GSNO treatment.

It was shown by Fan et al. (2012) that exogenous NO (50 µM sodium nitroprusside – SNP) could protect *Dendrobium huoshanense* against drought stress by increasing the demethylation ratio of genomic DNA regions methylated by stress. The other research group using the same MSAP (the methylation-sensitive amplified polymorphism) technique and gel-blot analyses presented DNA hypomethylation mainly at the CHG sites in correlation with transcriptional upregulation of genes and TEs in rice treated with high concentrations (0.5-1.0 mM) of SNP (Ou et al., 2015). However, excessive amounts of NO emitted from 0.5 mM SNP caused severe stress symptoms with inhibition of shoot and root growth in rice seedlings and complete silencing of the DNA chromomethylase 3 (*OsCMT3*) gene. In the same experiment, SNP exhibited hypermethylation in rice seedlings of two genotypes treated with a 50 µM (a 10-fold lower concentration).

Recently, it has been shown that transmethylation activity linked to TEs and stress-responsive gene expression is affected by GSNOR activity (Rudolf et al., 2021). GSNO reductase-deficient (*gsnor1*-*3*) *Arabidopsis* seedlings with a high NO level showed enhanced DNA methylation and reduced expression of TEs and stress-responsive genes compared with the wild type. The *Arabidopsis sahh1* knock-down mutant (S-adenosylhomocysteine hydrolase) with a decreased methylation index revealed enrichment of hypomethylated sites in defined genomic regions of the methylome.

Our study suggests that the high global 5-mC DNA levels in the following time points after potato inoculation were probably due to the inhibition of TEs and defense gene suppressors. Although the expression of TEs was not analyzed in this experimental approach, the previous study revealed a transient increase in mRNA transcript levels for the *NPR1*, *WRKY1*, and *PR1* key defense genes in a successful potato response to avr *P*. *infestans* (Drozda et al., 2022).

So far, no direct correlation between NO and DNA methylation has been fully confirmed in mammals; however, some data recognized an association between NO production or inducible nitric oxide synthase (iNOS) expression and DNA methylation level (Huang et al., 2012). When gastric cancer cells were treated with *Helicobacter pylori*, they showed enhanced NO synthesis, increased methyltransferase (DNMT) activity, and DNA methylation. The iNOS inhibitor (L-NAME) or demethylating agent returned both NO and methylation levels to the baseline. This documented that in the presence of NO-producing macrophages, *H*. *pylori*-induced epigenetic silencing of the tumor suppressor *runx3* gene *via* DNA methylation was reversed by treatment with a NOS inhibitor. Other experiments also revealed the gene-silencing effects through DNA methylation after SNP treatment of rat RINm5F cells, and here also, the outcomes were thoroughly arrested by the iNOS inhibitor (Hmadcha et al., 1999).

### SUVH4 mediated H3K9me2 functionally cooperates with DNA methylation in response to pathogen or GSNO

The main idea for *de novo* DNA methylation in plant resistance is the maintenance of plant genome stability by preventing TEs movement or blocking their binding to the specific 5-mC DNA sequences to avoid inhibition of defense genes by activating suppressor gene transcription (Dowen et al., 2012; Viggiano and de Pinto, 2017; Huang and Jin, 2021). The interaction of CMT3 and KYP/SUVH4 constitutes a self-reinforcing loop in repressive DNA methylation, while histone modification marks specify one another to maintain an epigenetic state (Pikaard and Scheid, 2014; West et al., 2014). The *KY*P gene mutation reduced CHG methylation, and the *CMT3* knockdown mutant revealed reduced histone methylation (Du et al., 2015; Wendte et al., 2019; Nozawa et al., 2021). Besides the direct link between CMT3 and KYP/SUVH4, an indirect association was also documented between SUVH4-mediated H3K9me2 and the RdDM pathway (Gouil and Baulcombe, 2016; Li et al., 2016).

In this study, GSNO treatment or pathogen-induced excessive amounts of NO caused enhanced DNA methylation correlated with a SUVH4-mediated high H3K9me2 level on the promoter *R3a* gene. When NO production and H3K9me2 declined (at 6 h), *R3a* gene transcript upregulation resulted. This finding indicates that reduced NO bioavailability, probably regulated by GSNOR, is pivotal in establishing potato resistance to the pathogen. The data confirmed our previous study since histone methylation is functionally linked to DNA methylation (Drozda et al., 2022).

Also, the GSNOR1-deficient mutant (*gsnor1-3)* with an elevated level of NO showed a significant increase in the global H3K9me2 level, in contrast to *sahh1* plants resulting in loss of the H3K9me2 mark (Rudolf et al., 2021).

Mammal cancer cells exposed to either DETA/NO and cellular sources of NO demonstrated changes in H3K9 methylation patterns, which is considered a gene-silencing mark (Hickok et al., 2013). The level of H3K9me2 became enriched around the promoter regions of most genes that were downregulated by tumor-associated NO overproduction, suggesting a causal link between the change in histone PTMs and altered gene expression related to the progression of more aggressive cancers (Hickok et al., 2013; Vasudevan et al., 2015).

There is evidence that NO exerts its regulatory function on DNA methylation and gene expression *via* S-nitrosylation of the enzyme engaged in maintaining a proper cellular methylation state. It has been demonstrated in *Arabidopsis thaliana* that methionine synthase (MS), S-adenosyl methionine synthase (SAMS), or S-adenosylhomocysteine hydrolase (SAHH) are affected by the reaction of NO with reactive thiols in cysteine (Cys) residues (Lindermayr et al., 2005; Abat and Deswal, 2009; Hu et al., 2014; Puyaubert et al., 2014; Hu et al., 2015). In addition to S-nitrosylation, crucial components of the SAM/SAH ratio undergo Tyr-nitration, as SAHH was found earlier in sunflower (Chaki et al., 2009) and potato (Arasimowicz-Jelonek et al., 2016). Also, in this study Tyr-nitration of SAHH in inoculated potato correlated in time (at 6 hpi) with the inhibition of *SAHH* transcript accumulation.

### Genes of the RdDM pathway responsive to nitric oxide are involved in potato immunity to late blight

Stress-induced *de novo* DNA methylation controlled by the RdDM pathway involves many regulators and primarily targets heterochromatic regions enriched with TEs and DNA repeat sequences (Cai et al., 2019; Huang et al., 2019). The functional relevance of individual methyltransferase/demethylase that controls plant immunity remains largely unknown, mainly due to the complex network and crosstalk between the different modulators that regulate resistance gene expression.

Our experiment exploring the *de novo* methylation pathway *via* the RdDM in disease regulation revealed an indirect link between NO and miRNAs, influencing the translation of target *R* genes. We found an opposite expression profile of *miR482e* and its target, the *R3a* gene involved in potato (cv. Sarpo Mira) immunity to late blight. This indicates that the transient decrease (at 6 hpi) in NO generation and downregulation of *DCL3* and *AGO4* diminished *miR482e* gene expression and allowed upregulation of the *R3a* gene. Notably, the following NO-responsive *miR6026* targets *Rpi*-*phu1* to trigger resistance response to avr *P*. *infestans* in the TG line has shown a similar effect. Consistently with these findings, also GSNO treatment provoked a time-dependent negative correlation between *R* and miRNA gene expression in both potato genotypes.

Recently published results showed that exogenous NO (SNP) could induce miRNAs in *Medicago sativa* plants subjected to drought stress (Zhao et al., 2019). The authors assumed that NO-sensitive miRNA downregulated transcription might play a positive regulatory role in drought stress response. As a result of deep sequencing analysis, it found that 24 known miRNAs and 31 novel miRNAs responded to NO under stress. Some of the exogenous NO reactive miRNAs targeted stress-responsive genes with the opposite expression profiles were engaged in enhanced drought tolerance. SNP-induced miR156 or miR399 downregulation enabled the synthesis of anthocyanins or positively regulated phosphate homeostasis in alfalfa responses to drought stress (Zhao et al., 2019). Among differentially expressed miRNAs that were explicitly induced or silenced by exogenous NO, the following miRNAs target genes or proteins, e.g., miR2513-5p (disease resistance protein), miR7696a-5p (chitin-binding; protein kinase) and miR398a-5p (Cu/Zn-superoxide dismutase copper chaperone) deserve special attention (Cohu et al., 2009; Devers et al., 2011; Eyles et al., 2013; Zhao et al., 2019).

An exciting relationship was found between NO and miRNA in controlling apoptosis in mammals (Lee et al., 2015). The NO-donor treatment (SNP) upregulated miR-1, which targeted Hsp-70, triggering apoptosis in osteoblasts. Moreover, the link between NO and the differential expression of numerous miRNAs was previously documented in the progression of various cancers and inflammatory diseases (de la Cruz-Ojeda et al., 2021). Differentiated upregulation of NOS expression is closely linked to antitumoral or oncogenic properties of nitric oxide, which are affected by multiple factors.

Importantly, our findings revealed that *DICER* (*DCL3*), *ARGONAUTE* (*AGO4*), and *DRM2* genes, the main components driving DNA methylation mediated by siRNAs, showed similar time-dependent profiles of transcriptional activity correlated with opposite miRNA/*R* gene expression and HR resistance to *Phytophthora infestans*.

In *Arabidopsis thaliana*, silencing of *AGO4* leads to increased susceptibility to the virulent bacterial pathogen *Pseudomonas syringae* (Agorio and Vera, 2007; Yu et al., 2013). Also, other RdDM mutants, including *nrpe1*, *nrpd2*, *ago4*, *drd1*, and *rdr2*, showed reduced resistance to *P*. *syringae* (López et al., 2011), which indicates the critical importance of the RdDM pathway and TEs targeted specifically for DNA methylation in the regulation of plant immunity.

Recently reported in animals is a fascinating example of a potential mechanism for microbiota-dependent miRNA-based regulation of host gene silencing by NO-mediated S-nitrosylation (Seth et al., 2019). The authors have documented that S-nitrosylation of AGO2 in a nematode by NO derived from the microbiota-inhibited miRNA targets of *C*. *elegans.*

### Transient upregulation of *ROS1* correlates with potato *R3a* gene expression

DNA demethylases possess the Fe-S binding motif as their cofactor essential to catalyze the excision of 5-methylcytosine, followed by cytosine replacement through the base excision repair pathway. Under biotic stress conditions, various redox components, including NO, can alter DNA demethylation, disrupting the Fe-S cluster and repressing demethylase activity (Vasudevan et al., 2016; Socco et al., 2017).

Studies, which have been conducted for many years on mammals, provide essential insights into how NO can inhibit mononuclear non-heme iron dioxygenases enzymes, such as histone Jumonji C demethylases (JMJC) and DNA demethylase (Ten Eleven Translocation-TET) by producing a nitrosyl-iron complex in the active pocket of the enzyme or *via* formation of dinitrosyliron complexes (DNICs) that reduce the iron cofactor availability (Hickok et al., 2013; Cheng et al., 2014; Bovee et al., 2018; Palczewski et al., 2019). It was documented that a NO donor (DETA/NO) could inhibit the catalytic activity *in vitro* and the expression level of JMJC domain-containing histone demethylase (KDM3A) in a dose-dependent manner (Hickok et al., 2013). A similar effect was found when TET enzyme activity significantly decreased in cancer cells exposed to NO, supported by EPR studies showing that NO could directly bind to catalytic non-heme iron (Bovee et al., 2018).

ROS1, instead of TET, regulates plant developmental and stress responses (Gong et al., 2002), and its expression is influenced by the activity of the RdDM and active DNA demethylation pathways. The *ROS1* promoter in *Arabidopsis* contains a DNA methylation monitoring sequence (MEMS) that functions as an indicator to sense DNA methylation levels and regulates DNA methylation by controlling *ROS1* expression (Zhu, 2009; Lei et al., 2015; Williams et al., 2015).

In this study, the transcript level of ROS1 drastically increased soon after *DRM2*, *DCL3*, and *AGO4* genes reached the maximum of their expression, demonstrating a tight interconnection with the RdDM pathway and facilitating *R3a* gene upregulation. Our research shows that *ROS1* might counteract the DNA methylation pathway to prevent *R3a* gene silencing in potato exposed to GSNO or avr *P*. *infestans*.

Methylation-sensitive regulation of *ROS1* expression is robustly down-regulated in DNA methylation-defective mutants. The triple DNA demethylase *Arabidopsis* mutant (*ros1 dml2 dml3*) showed enhanced susceptibility to *Fusarium oxysporum* (Le et al., 2014). In turn, a hyper-methylated *ros1* mutant of *Arabidopsis*, which is affected in DNA demethylation, displayed enhanced susceptibility towards Pto DC3000 and attenuated resistance to *Hyaloperonospora arabidopsis*, in contrast to a hypomethylated *nrp1* mutant with impaired RdDM methylation (Yu et al., 2013; López Sánchez et al., 2016). Recently it was found that ROS1 positively regulates basal resistance towards Pto DC3000 by counteracting RdDM activity (Halter et al., 2021). Cited authors documented that ROS1, by demethylating the *RMG1* (functional disease resistance gene) promoter, antagonizes DCL2 or DCL3 functions and facilitates proper flg22-triggered induction of this gene.

There is weak evidence for a direct NO effect on active DNA demethylation mechanisms in plants. However, NO may indirectly influence the demethylation process by forming DNIC or constitute complexes with iron-sulfur-containing proteins and non-heme iron proteins that affect their activity. The microarray analyses of *nia1nia2* with a decreased NO level showed *ROS1* upregulation (Gibbs et al., 2014). The infiltration of *Arabidopsis* leaves with 1 mM CysNO resulted in the downregulation of *ROS1* (Hussain et al., 2016).

Our experiment showed that other demethylase (*StDME* and *DML*-*like*) genes were rather not responsive to GSNO or pathogen treatment. Still, it may not be excluded that the increase in the global 5-mC DNA level in the presence of NO was due to an inhibition of demethylase activity coded by these genes.

## Conclusions

Our conclusions are based on the results obtained from the potato leaves exposed to biological (NO burst) or chemical (GSNO) NO sources. When comparing the effects, we found similarities and differences based on the NO origin, partially confirming the competency of NO signaling to affect expression profiles of DNA methylation/demethylation genes under stress. In potato response to avr *P*. *infestans*, the emphasis was placed on the timing and intensity of biphasic NO generation during NO burst, which influenced changes in *de novo* DNA methylation processes. This finding aligns with our previous concept postulated that biphasic NO production, downregulated by GSNOR activity, is required to trigger histone modifications and reprogram the transcriptional network of potato defense genes to avr *P. infestans* (Drozda et al., 2022).

**Figure 8:**
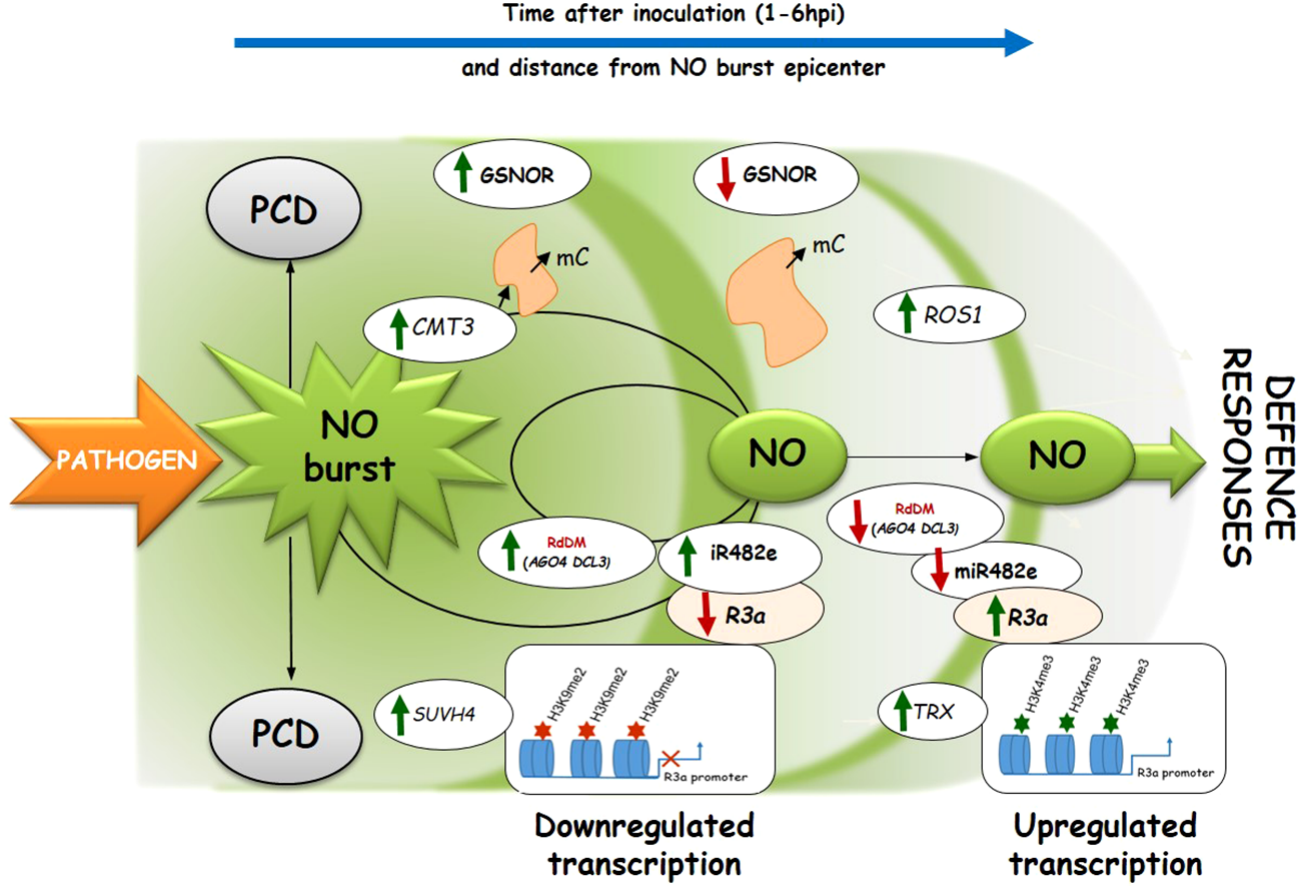
The proposed scheme illustrates the NO signaling effects on genes regulating DNA (de)methylation, being in dialog with histone methylation in potato leaves challenged with avr *P. infestans*. Pathogen-induced NO generation diminishes in the following hours after inoculation (at 1-6 hpi) and at further distances from the epicenter of the NO burst. Pathogen-induced NO signaling (at 3 hpi) promotes DNA methylation and upregulation of the RdDM pathway genes (*DCL3*, *AGO4*, *DRM2*, and *miR482e*), suppressing the *R3a* gene having high H3K9me2 level on its promoter. A drop in NO bioavailability (at 6hpi) due to GSNOR activity (Drozda et al., 2022) results in the reduced inhibitory effect of the *miR482e* toward the corresponding *R3a* gene (having a high H3K4me3 level on its promoter), which favors potato resistance to *P. infestans*.

This paper shows functional interconnections between SUVH4-mediated H3K9me2 and DNA methylation under controlled NO levels in potato response to biotic stress. A timely decrease in NO bioavailability revealed a negative correlation between downregulated miRNAs and upregulated target *R* genes, favoring the resistance of two potato genotypes to late blight (**Figure 8**). Hopefully, future research will expand our patchy knowledge about epigenetic mechanisms including the NO-signaling in plant immunity to stress.

## Data availability statement

The original contributions presented in the study are included in the article/**Supplementary Material**. Further inquiries can be directed to the corresponding author.

## Author contributions

AD: Performing all plant and pathogen experiments, data analysis of gene expression. BK and AD: ChIP analysis and potato *in vitro* culture. YG: Performing NO detection and participation in *R* and miRNA gene expression. MA-J: Conceptualization, writing – review, and editing. JP: TG line culture and analysis. PJ: Western blot analysis. DK: Participation in statistical analyses. JF-W: Conceptualization, writing – original draft preparation, writing – review and editing with contributions of all the authors. All authors have read and agreed to the published version of the manuscript. All authors contributed to the article and approved the submitted version.

## Funding

This research was funded by the Polish National Science Centre; project NCN No. 2017/25/B/NZ9/00905; The publication was co-financed within the framework of the Polish Ministry of Science and Higher Education’s program: “Regional Initiative Excellence” in the years 2019– 2022 (No. 005/RID/2018/19).

## Conflict of interest

The authors declare that the research was conducted in the absence of any commercial or financial relationships that could be construed as a potential conflict of interest.

## Publisher’s note

All claims expressed in this article are solely those of the authors and do not necessarily represent those of their affiliated organizations, or those of the publisher, the editors and the reviewers. Any product that may be evaluated in this article, or claim that may be made by its manufacturer, is not guaranteed or endorsed by the publisher.
