# Supplementary material for: Insights into the expression of DNA (de)methylation genes responsive to nitric oxide signaling in potato resistance to late blight disease
Source: Front Plant Sci. 2022 Dec 2;13:1033699. doi: 10.3389/fpls.2022.1033699 (PMC9815718; doi:10.3389/fpls.2022.1033699)
Supplement: Supplementary file 1 [file DataSheet_1.docx]

**Table S1.** List of designed primers for RT-qPCR.

| Gene | Sequence (5’->3’) | | Accession Number | Tm [°C] |
| --- | --- | --- | --- | --- |
|  | Forward | Reverse |  |  |
| *ef1α* | ATTGGAAACGGATATGCTCCA | TCCTTACCTGAACGCCTGTCA | AB061263 | 53 |
| *18s rRNA* | GGGCATTCGTATTTCATAGTCAGAG | CGGTTCTTGATTAATGAAAACATCCT | X67238 | 60 |
| *SAHH* | CATGGCTTCCCGTACTGAAT | TCACCTTTCCAGGCAAAAAC | DQ252503.1 | 58 |
| *CMT3* | TTGTGACGAGAGCTGAACCC | GGAAAACCTTGGAGCCTTGC | XM_004252792.3 | 60 |
| *DRM2* | AAGTTGGGGTTCAGTGTTTGC | TGTGCCACAGCAAAAGCATA | XM_015312485.1 | 59 |
| *StDME* | GTCGAACGGCAAATAGGGGA | GGCCCTTGCAACAGGAATTG | PGSC0003DMT400023712 | 58 |
| *DME-like* | TGTTCTTCTTGCAGGTCCCG | TGTTCTGGTTCTGGCCCATC | XM_006351291 | 56 |
| *JMJ706* | TTATCAGCACTGTGGGGCAG | CACATGCTCTCGGACAACCT | XM_006352846.2 | 53 |
| *ROS1* | GGGGTTTTTGGTTCCTCAAT | TCAAGCCCATGCTATTACCC | XM_015314854 | 57 |
| *DCL3* | GCCACTGATGTGGTTGAGGA | GAGCACGTCCCCTAGATTGG | XM_006361458 | 60 |
| *AGO4* | CTGGGCCTATCGTGGACTTC | GGCTCGTCTTCACCCTCAAA | XM_006362679 | 60 |
| *SUVH4* | TCCAAGAGTTTGTTCTTGTGCT | ACTGTAAGGCAACTTCCACCA | XM_015307858.1 | 53 |
| *R3a* | AGCCAACTGGTGAGATTAGT | ATCCTGTACCCACAATTTGC | PGSC0003DMG400009455 | 53 |
| *Rpi-phu1* | AGAGACCCTGGATATATTTCATAGCTCT | CGCTCTAGGCACAGGGCTCAATGCTGAT | FJ423044.1 | 50 |

| Primer | Sequence (5’->3’) | Tm [°C] | Accession Number | Stage |
| --- | --- | --- | --- | --- |
| *miR482e RT* | GTCGTATCCAGTGCAGGGTCCGAGGTATTCGCACTGGATACGACTTGGAA | - | MI0020245 | Reverse transcription |
| *miR482e F* | TCTTGCCAATACCGCCCAT | 56 |  | RT-qPCR |
| *miR482e R* | ATCCAGTGCAGGGTCCGAGG | 58 |  |  |
| *miR6026 RT* | GTCGTATCCAGTGCAGGGTCCGAGGTATTCGCACTGGATACGACGCAATA | - | MI0020256 | Reverse transcription |
| *miR6026 F* | TCGCTTTCTTGGCTAGAGTTG | 57 |  | RT-qPCR |
| *miR6026 R* | ATCCAGTGCAGGGTCCGAGG | 56 |  |  |

**Table S2.** List of designed primers for quantitative stem-loop PCR.

**Table S3.** List of designed primers for ChIP-qPCR (promotor sequence) analyses.

| Gene | Sequence (5’->3’) | | Accession Number | Tm [°C] | Promotor region sequence +500bp |
| --- | --- | --- | --- | --- | --- |
|  | Forward | Reverse |  |  |  |
| *R3a* | AGCCAACTGGTGAGATTAGT  Start: 240 Stop: 260 | ATCCTGTACCCACAATTTGC  Start: 382 Stop: 363 | PGSC0003DMG400009455 | 56 | ttctgaaatctgcaaagcaaaacacaaaatgattttaagaaatttgagaagatgaatgtt  tctatcttcaattactaacaactttactttacctgttgaatgctaagagtaatttgcaaa  caagaatgatcactgatttctctctctcttttgctagtaaatttgcaaacaacaagttgt  aactatgtttgctagtgcctattcattgctttctcaattgagtaattgagggtagttggg  ggacgttgtacaaaatggggaccatttagacaccaaagtcttgtatcatttaaacatcaa  tacaaggttcaattgtgctattttgacatttttggctaattcagccaaatacgttaaagt  gtgcaacacactcgcggataacatctcaatatttgaattgcacaaaatgacaaaattact  tccatatttgaattgcacaaattactttcctggggtgtttgacaattaatgagtcctgtt  ttaaggcttgtagtccttatt |
