# Supplementary figures and images for: Insights into the expression of DNA (de)methylation genes responsive to nitric oxide signaling in potato resistance to late blight disease
Source: Front Plant Sci. 2022 Dec 2;13:1033699. doi: 10.3389/fpls.2022.1033699 (PMC9815718; doi:10.3389/fpls.2022.1033699)

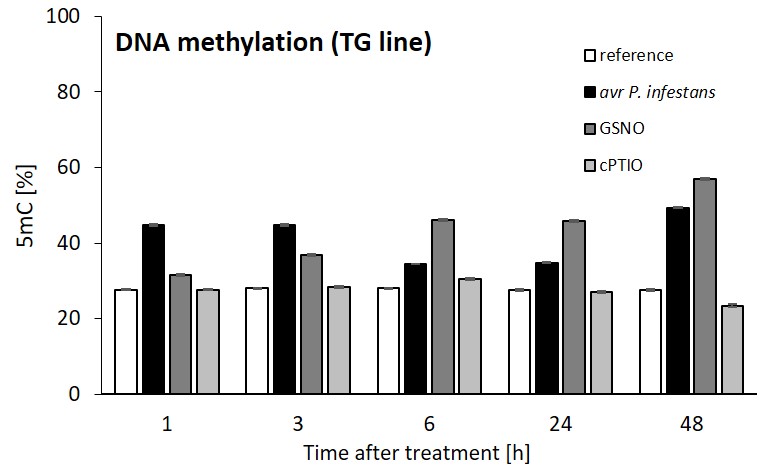

Supplement: Supplementary file 2 [file Image_1.jpeg]

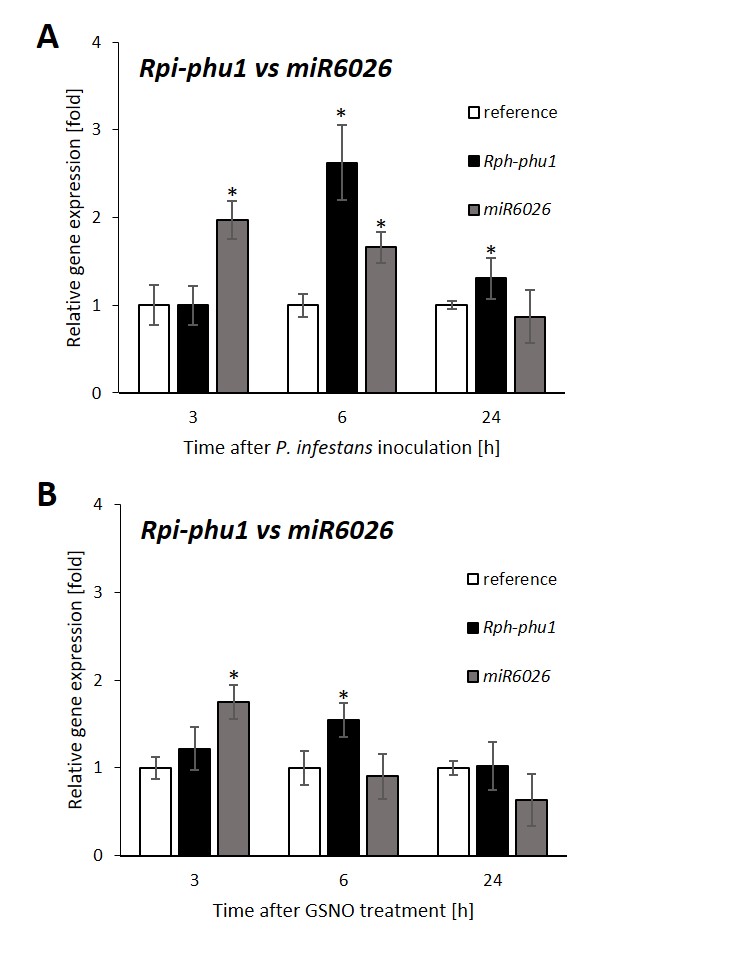

Supplement: Supplementary file 3 [file Image_2.jpeg]

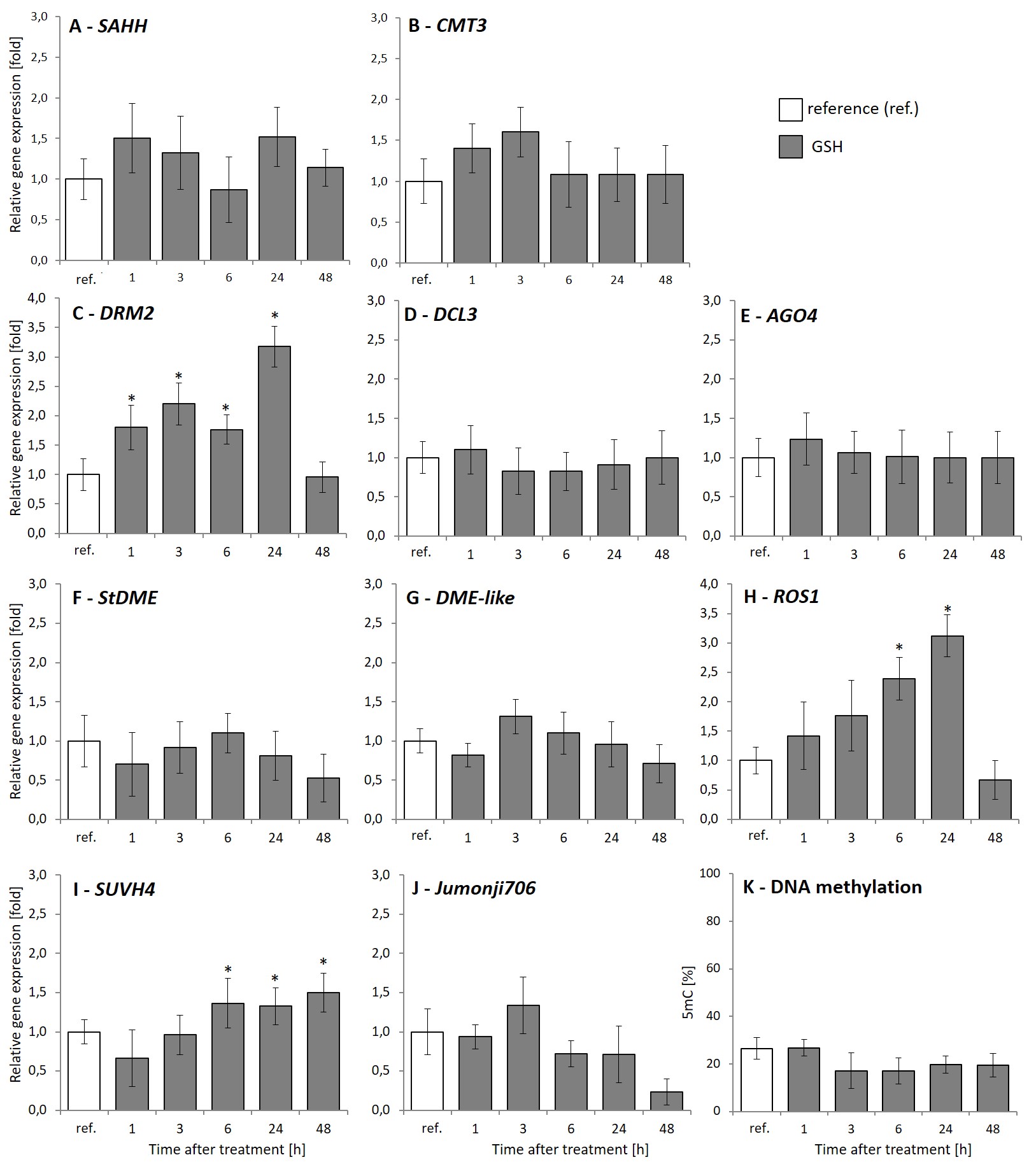

Supplement: Supplementary file 4 [file Image_3.jpeg]

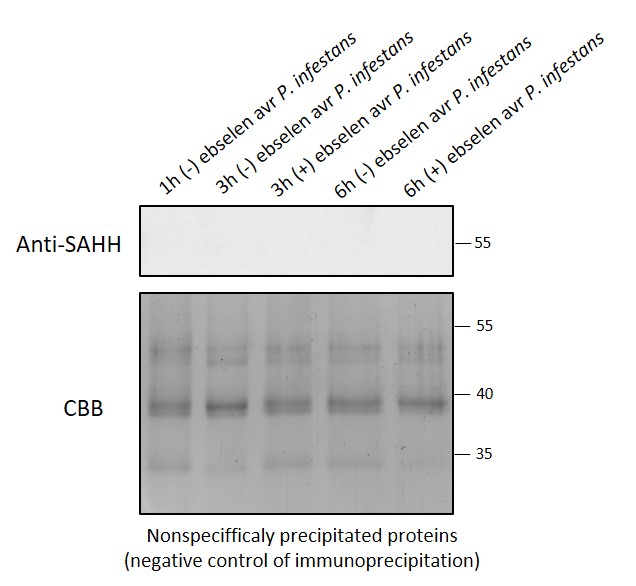

Supplement: Supplementary file 5 [file Image_4.jpeg]

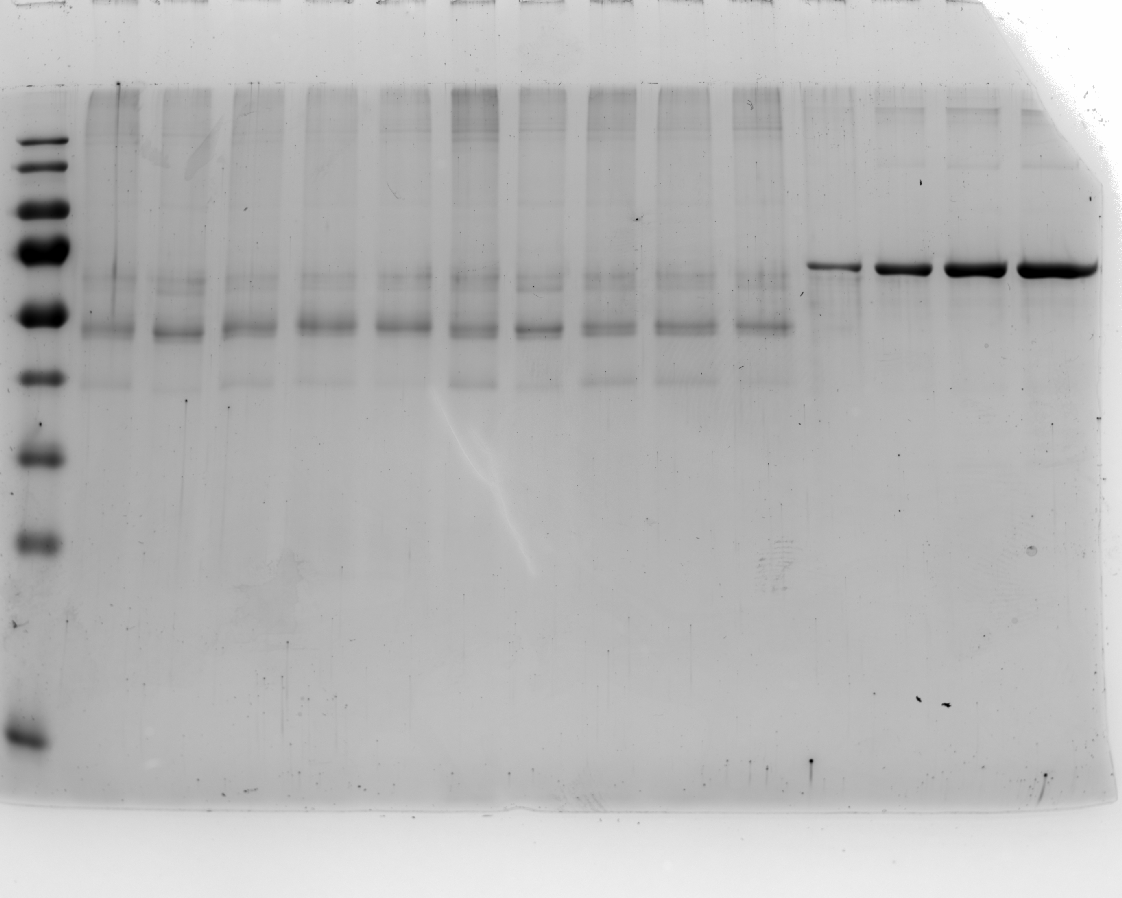

Supplement: Supplementary file 6 [file Image_5.tif]

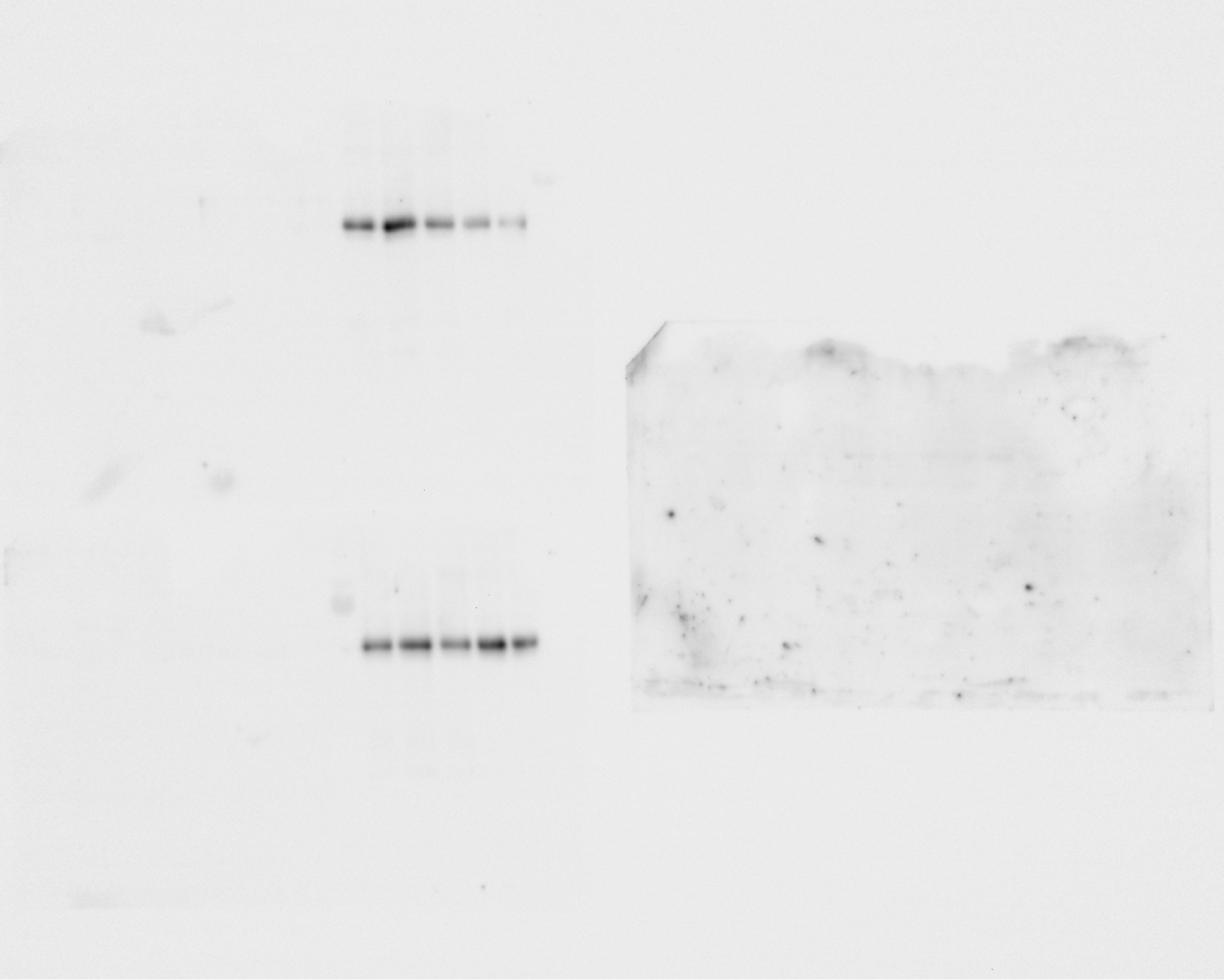

Supplement: Supplementary file 7 [file Image_6.tif]
